# Supplementary material for: Dietary Complex and Slow Digestive Carbohydrates Promote Bone Mass and Improve Bone Microarchitecture during Catch-Up Growth in Rats
Source: Nutrients. 2022 Mar 19;14(6):1303. doi: 10.3390/nu14061303 (PMC8951765; doi:10.3390/nu14061303)
Supplement: Supplementary file 1 [file nutrients-14-01303-s001.zip › nutrients-1618144-supplementary.pdf]

Supplementary material

Table S1. Bone densitometry parameters at the end of the restriction period

Densitometry parameters	Tibia		Femur		Lumbar Vertebrae (2-5)	
	NR	RR	NR	RR	NR	RR
BMD (mg/cm^2)	158.2 ±2.33	127.4 ±6.61 *	214.6 ±7.02	160.2 ±13.16 *	167.7 ±4.27	119.7 ±9.18 *
BMC (g)	0.3049 ±0.01	0.1688 ±0.22 *	0.3537 ±0.02	0.2437 ±0.04 *	0.3089 ± 0.015	0.1484 ±0.03 *

Data presented as mean ±S.E.M. NR, not restricted group; RR, restricted group; BMC, bone mineral content; BMD, bone mineral density. *Significant difference compared to the NR group (p-value <0.05).

Table S2. Micro-CT trabecular parameters of tibia and vertebra at the end of the restriction period

Micro-CT Parameter	Tibia		Vertebra	
	NR	RR	NR	RR
BV/TV (ratio)	0.1207 ±0.011	0.045 ±0.007*	0.277 ±0.010	0.233 ±0.010*
Tb.Th (mm)	0.051 ±0.001	0.050 ±0.003	0.077 ±0.001	0.066 ±0.002*
Tb.N (1/mm)	3.329 ±0.267	1.566 ±0.214*	3.777 ±0.101	3.593 ±0.046
Tb.Sp (mm)	0.275 ±0.017	0.6980 ±0.095*	0.244 ±0.008	0.258 ±0.004
Conn.D ($1/mm^3$)	83.32 ±9.326	23.22 ±5.215*	93.69 ±4.446	94.75 ±3.432

Data presented as mean ±S.E.M. NR group, not restricted group; RR group, restricted group; BV/TV, bone volume/total volume; Tb.Th, trabecular thickness; Tb.N, trabecular number; Tb.Sp, trabecular separation; Conn.D, connectivity density. *Significant difference compared to NR group (p-value <0.05).

Table S3. Micro-CT growth plate parameters at the end of the restriction period

Micro-CT Parameter	NR	RR
GP Surface (mm^2)	46.62 ±1.410	30.87 ±1.605*
GP Thickness (mm)	0.206 ±0.009	0.133 ±0.014*
GP Volume (mm^3)	7.049 ±0.568	2.758 ±0.611*

Data presented as mean ±S.E.M. NR group, not restricted group; RR group, restricted group; GP, growth plate. *Significant difference compared to NR group (p-value <0.05).

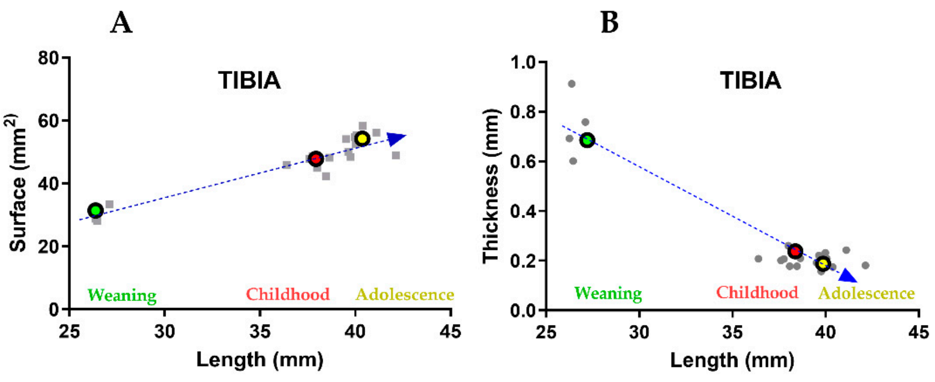

Figure S1. Evolution of tibia growth plate surface (**A**) and thickness (**B**) in normal fed rats throughout the different stages of life (NR group).
